# Effect of electroacupuncture in postanesthetic shivering during regional anesthesia: a randomized controlled trial

**DOI:** 10.1186/1472-6882-12-233

**Published:** 2012-11-27

**Authors:** Bo-Yan Yeh, Yi-Chun Hsu, Jyun-Yan Huang, I-Ting Shih, Wei-Jia Zhuo, Yung-Fong Tsai, Chee-Jen Chang, Huang-Ping Yu

**Affiliations:** 1 Department of Chinese Acupuncture and Traumatology, Center for Traditional Chinese Medicine, Chang Gung Memorial Hospital, Taoyuan, Taiwan; 2 Department of Anesthesiology, Chang Gung Memorial Hospital, 5 Fu-Shin Street, Kwei-Shan, Tao-Yuan 333, Taiwan; 3 Department of Chinese Internal Medicine, Center for Traditional Chinese Medicine, Chang Gung Memorial Hospital, Taoyuan, Taiwan; 4 Biostatistical Center for Clinical Research, Chang Gung Memorial Hospital, Taoyuan, Taiwan; 5 College of Medicine, Chang Gung University, Taoyuan, Taiwan

**Keywords:** Acupuncture, Shivering, Thermoregulation

## Abstract

**Background:**

Shivering during regional anesthesia is a common complication and is related to a decrease in the patient’s core body temperature. Previous studies have shown that acupuncture on specific acupoints can preserve core body temperature. The present study evaluated the effect of electroacupuncture in preventing the shivering caused by regional anesthesia.

**Methods:**

This prospective and randomized controlled study analyzed the data from 80 patients undergoing urological surgery, who were classified as ASA I or II. Spinal anesthesia was performed in all patients using 15 mg of bupivacaine. The patients were randomly allocated to receive either placebo acupuncture (Group P, *n* = 40) or electroacupuncture (Group A, *n* = 40) for 30 min before administration of spinal anesthesia. Shivering score was recorded at 5 min intervals, with 0 representing no shivering and 4 representing the most severe shivering possible. Heart rate, blood pressure, and tympanic temperature were recorded before the intrathecal injection, and again every 5 min thereafter until 30 min.

**Results:**

After spinal anesthesia, the decrease in tympanic temperature was less for Group A patients than Group P, with the difference being statistically significant. After 15 min, 13 patients in Group P attained a shivering score of 3 or more, compared with 3 patients in Group A. Significantly more patients in Group P attained a shivering score of at least 1.

**Conclusions:**

The prophylactic use of electroacupuncture might maintain core body temperature, and may effectively prevent the shivering that commonly develops during regional anesthesia.

**Trial registration:**

Australian New Zealand Clinical Trials Registry ACTRN12612000096853.

## Background

Regional anesthesia may impair a patient’s thermoregulatory control [1], with associated shivering being reported in up to 64% of cases [2-4]. Shivering during neuraxial anesthesia may potentially have a detrimental effect [5]. Various opioid and non-opioid pharmacological treatments are used to manage postanesthetic shivering, but several side-effects have been reported for these agents, including hypotension, hypertension, sedation, respiratory depression, nausea, and vomiting [4,6-9].

Acupuncture has been reported to exert a beneficial effect on postanesthetic nausea and vomiting [10]. However, the effectiveness of acupuncture as a prophylactic modality against postanesthetic shivering had not been investigated. Regional anesthesia produces vasodilatation, which facilitates core-to-peripheral redistribution of heat and decreases the core body temperature [11]. Several studies have reported that acupuncture exerts a thermoregulatory effect, but the mechanisms were not entirely understood [12,13]. Acupuncture applied to a specific acupoint may induce slight core hyperthermia with a decrease in skin temperature, a finding which suggests that acupuncture might be able to reverse the core-to-periphery redistribution of heat [13].

Electroacupuncture rather than manual acupuncture has been used in many studies, because the parameters of electroacupuncture can be precisely controlled, allowing the study to be replicated [14]. We hypothesized that electroacupuncture intervention might be able to prevent postanesthetic shivering. To test this hypothesis, we examined the effect of electroacupuncture treatment on postanesthetic shivering during regional anesthesia in patients undergoing ureteroendoscopic surgery.

## Methods

### Patient selection

The study was approved by the Institutional Review Board of Chang Gung Medical Foundation and Australian New Zealand Clinical Trials Registry. Informed consent was obtained from every participant. A total of 85 patients of both sexes were prospectively included in the study. All patients were scheduled for elective ureteroendoscopy (URS) surgical procedures under spinal anesthesia between June 2010 and May 2011. We excluded from the study patients who had previously received acupuncture; or who had a history of hypo- or hyperthyroidism, cardiopulmonary disease, or psychological disorder; or who needed blood transfusion during surgery; or whose initial body temperature was >38.0°C or <36.0°C; or who had a known history of alcohol or substance abuse; or who were receiving vasodilators, or medications likely to alter thermoregulation; or who had received an ASA (American Society of Anesthesiologists) classification equal to or greater than 3; or who were younger than 20 years or older than 80 years.

### Study setting

All participants were randomly assigned to either Group A (electroacupuncture) or Group P (placebo) on the basis of a concealed allocation approach. A computerized random number table was used to determine the allocation to groups, with numbered opaque sealed envelopes containing the randomization schedule, and no restrictions on the randomization. The envelopes were kept by an investigator who was not an assessor of the study. The envelopes were opened immediately before the electroacupuncture treatment and acupuncturists were informed by messages sent by the investigator. Two groups of health care personnel participated in the study. The sealed envelopes were accessible only to the first group, consisting of acupuncturists who were to perform the electroacupuncture. The second group, namely anesthetists who were to perform spinal anesthesia, were blinded to the electroacupuncture allocation. All patients were also blinded to their allocation. Data were collected by nursing staff who were unaware that an electroacupuncture study was being conducted.

### Electroacupuncture and placebo settings

Half of the patients in our overall sample were allocated to receive placebo acupuncture intervention (Group P, *n* = 40), and the other half received active electroacupuncture intervention (Group A, *n* = 40). All patients received the treatment or placebo treatment for 30 min before spinal anesthesia was administered. Two Taiwan-trained and licensed acupuncturists with a median of 4 years of experience provided study treatment in operating room at Chang Gung Memorial Hospital, Linkou. Group A patients received acupuncture at the ST36 (Zusanli) and ST37 (Shangjuxu) points bilaterally, with electrical stimulation, for 30 min before anesthesia. 30 G 3.8 cm long unused sterile needles were used for the study (YU KUANG, TAIWAN). Needles were inserted vertically to a depth not exceeding 1.3 cm. As the appropriate depth was reached, the acupuncturist did not manipulate the needles with twirling or lifting-inserting actions, but simply waited for *de qi,* which is the state of connection between the needle and the patient’s energetic flow. An electrostimulator, NihonRiko TOKKI MODEL III (NihonRiko Medical Co., Ltd., Nagasaki, Japan) was connected to the needles with a current of 1 mA and a frequency of 3 Hz. At this current intensity, patients typically feel a slight but not uncomfortable twitching. Electroacupuncture was applied for 30 min.

The control intervention (Group P) consisted of needling at non-channel points, with sham electrostimulation. For control patients, the procedures imitated those used for the active electroacupuncture intervention, but 1) needles were inserted at 4 points remote from any classically described meridian or extraordinary acupoint; and 2) no current was applied to the needles. The 4 points were located 3 cm lateral to the ST36 and ST37 points, respectively, bilaterally. The needles were inserted obliquely to a depth of approximately 1 cm, which was the minimum needed to allow a stable connection to the electroacupuncture leads. We used a control electrostimulator identical to the one that provided the electroacupuncture intervention, with the lead modified to prevent administration of electrical stimulation when the stimulator was turned on [15].

### Surgical environment during anesthesia

The temperature of the operating room was maintained at 24°C. Two liters of irrigation fluid were used during each operation. The irrigation and intravenous fluids were preheated to 37°C in a warmed cabinet, and administered without in-line warming. No other warming device was used. Each patient was covered with one layer of surgical drapes and one layer of a cotton blanket, positioned over the thighs and calves. In addition, one layer of a cotton blanket was placed over the chest and arms. Before the spinal anesthesia procedure, each patient received 10 ml/kg lactated Ringer’s solution. Subarachnoid anesthesia was instituted at the interspace of either L3/4 or L4/5. Hyperbaric bupivacaine (5 mg/ml, 15 mg) was injected using a 25 G Quincke spinal needle (B. Braun Melsungen AG, Melsungen, Germany).

### Measurement of blood pressure, heart rate, O_2_ saturation, and level of sensory block

Patients did not receive premedication. Their heart rate, blood pressure, and pulse oximetry arterial O_2_ saturation were recorded using standard non-invasive monitors before the intrathecal injection. Thereafter the same measurements were taken at 5, 10, 15, 20, 25, and 30 min respectively. During the perioperative period, sensory block was assessed with a pinprick test at 5 min intervals.

### Measurement of core body temperature

Before the intrathecal injection, and thereafter at each 5 min interval during the perioperative period, the patient’s core body temperature was monitored by assessing tympanic temperature. We used an infrared noncontact ear thermometer (Chang Gung Medical Technology Co., Ltd., Taipei, Taiwan) for this measurement.

### Evaluation of postanesthetic shivering

The presence and degree of shivering was recorded by an observer blinded to the study. Shivering was graded using a scale similar to that validated by Tsai and Chu [16], namely 0 = no shivering; 1 = piloerection or peripheral vasoconstriction, but no visible shivering; 2 = muscular activity in only one muscle group; 3 = muscular activity in more than one muscle group, but not generalized; and 4 = shivering involving the whole body. During surgery the patient’s shivering score was recorded at 5 min intervals. After the first 15 min, if a score of 3 or 4 was noted then the prophylaxis was regarded as having been ineffective, and 25 mg of intravenous meperidine was administered.

### Rescue method and side effects

Side effects such as hypotension, nausea, vomiting, and hallucinations were recorded. Hypotension was defined as a decrease in mean blood pressure of more than 20% from baseline; baseline mean blood pressure had been measured in the ward before surgery. This was treated by crystalloid infusion, and if necessary, 5 mg ephedrine was administered intravenously. The amount of ephedrine given in each case was recorded. If patients developed nausea or vomiting, 10 mg intravenous metoclopramide was administered. Hallucination as a side effect was defined as a false sensory experience where a patient reported seeing, hearing, smelling, tasting, or feeling something that was nonexistent.

The attending anesthetists also assessed the degree of sedation on a 5-point scale: 1 = fully awake and oriented; 2 = drowsy; 3 = eyes closed but rousable on command; 4 = eyes closed but rousable to mild physical stimulation; and 5 = eyes closed and unrousable to mild physical stimulation [17].

### Statistical analysis

The sample size was calculated using PASS 2008 software. We chose 15 cases in electroacupuncture group and 15 cases in control group for the pilot study. The timepoint for evaluation of poanesthetic shivering is 15 min after spinal anesthesia. The shivering rate is 60% in control group and 27% in electroacupuncture group. This number of cases per group would suggest an approximately 33% improvement in postanesthetic shivering. Consequently, a power calculation (α = 0.05 and β = 0.2) indicated that each group should include at least 35 patients. The SPSS program (v 17) was used to analyze the statistical data (SPSS Inc., Chicago, IL). All plots were performed by R 2.13.0 software.

Categorical variables were compared between the groups using the Chi-squared test and Fisher exact test, if the cases numbered fewer than 5 patients. Between-group data for continuous variables were analyzed using the *t*-test for 2 independent samples. The incidence of shivering and side-effects were compared using the Chi-square test and Fisher exact test, if the cases numbered fewer than 5 patients. The results were reported as mean (standard deviation). A *P*-value < 0.05 was considered statistically significant.

## Results

### Patient characteristics

**Figure 1:**
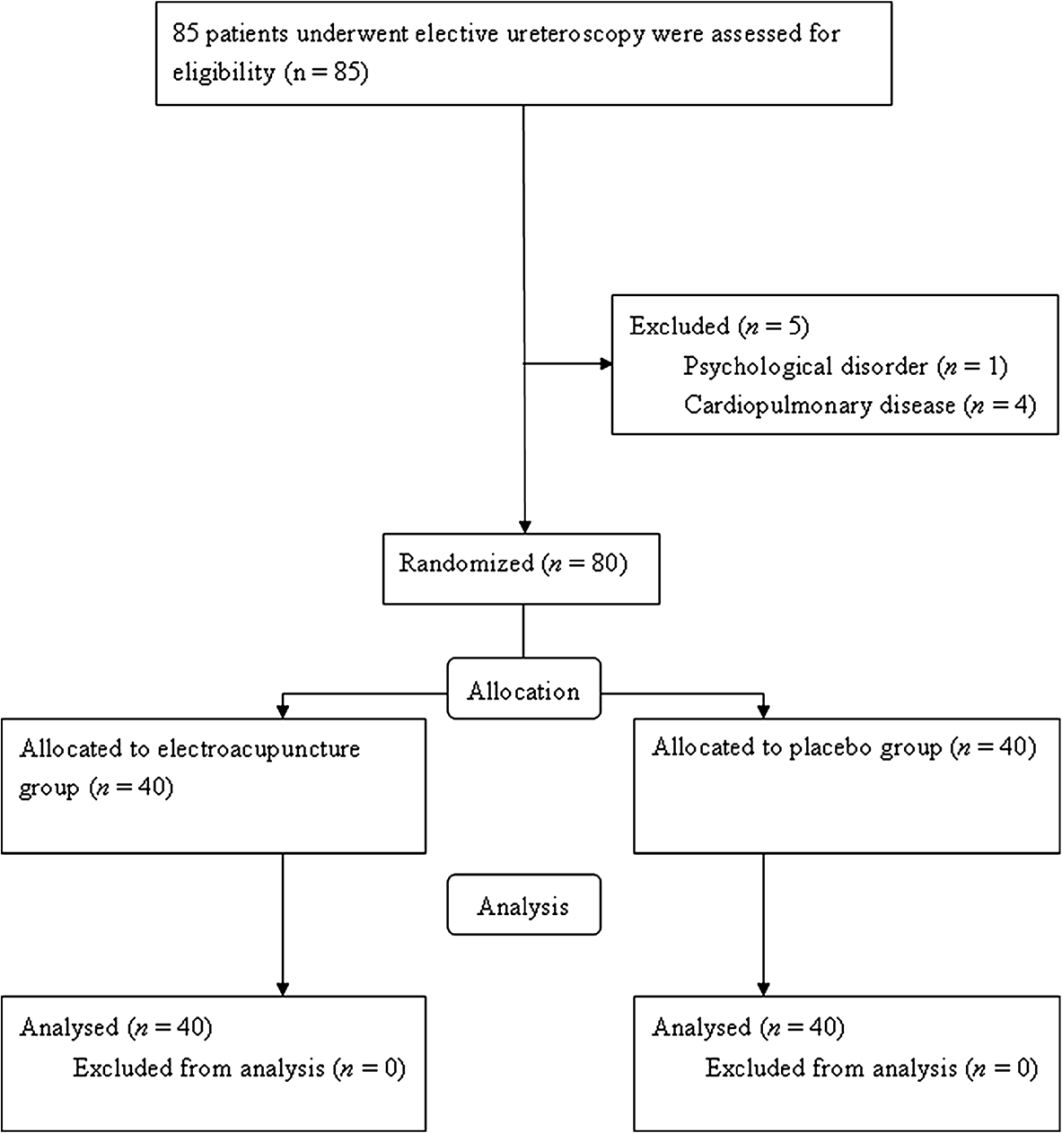
Consort flow diagram.

Eighty-five patients were enrolled for the study. One patient was excluded because of psychological disorder and 4 patients were excluded because of cardiopulmonary disease. The Consort flow diagram for the study is shown in Figure 1. Patient characteristics including sex, duration of surgery, and the median level of sensory block were similar among the groups (Table 1).

**Table 1:** Patients characteristics, duration of surgery, median level of sensory block, and incidence of shivering in the two groups

	**Group A**	**Group P**	***P-*value**
Number of patients	40	40	
Age (yr)	58.60 (11.92)	56.93 (14.62)	0.58
Gender (F/M)	7/33	3/37	0.18
Weight (kg)	67.81 (12.15)	69.01 (13.52)	0.68
Height (cm)	164.12 (6.76)	165.12 (6.67)	0.51
ASA (I/II)	19/21	13/27	0.17
Duration of surgery (min)	77.08 (40.72)	76.63 (39.47)	0.96
Median level of sensory block (dermatome)	T9 (T4-T12)	T9 (T4-L1)	

Data are presented as mean (SD) or median (range). The median level of sensory block was similar in both groups.

### Hemodynamic changes and side effects

**Figure 2:**
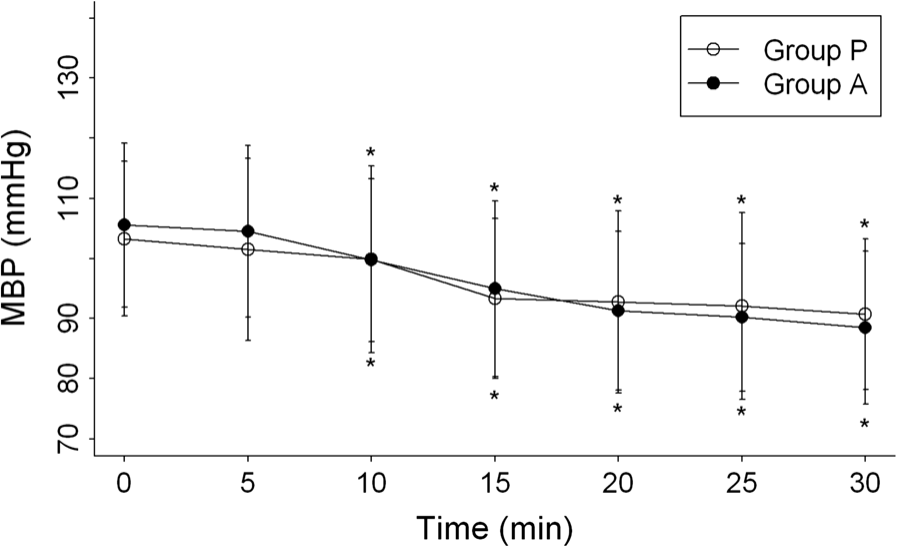
**Mean blood pressure (MBP) after spinal anesthesia within 30 min during the peri-operative period. **Group P = placebo group, Group A = electroacupuncture group. Data are shown as mean (standard deviation). *P < 0.05 compared with baseline within groups.

**Figure 3:**
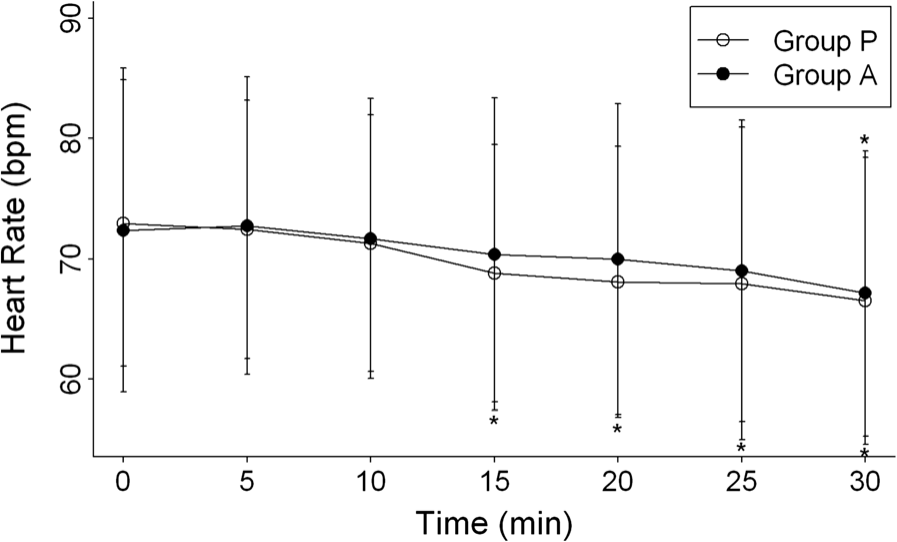
**Heart rate after spinal anesthesia within 30 min during the peri-operative period. **Group P = placebo group, Group A = electroacupuncture group. Data are shown as mean (standard deviation). *P < 0.05 compared with baseline within groups.

The sedation score was 1 in all patients just after intrathecal injection. No differences were observed between the groups for hemodynamic values (Figures 2 and 3). However, within-group differences were found for the comparison of preoperative and postoperative hemodynamic values (Figures 2 and 3). Hypotension developed in 12 patients in Group A and in 8 patients in Group P, but the difference between groups was not significant (Table 2). In all these patients, hypotension was successfully controlled by the administration of crystalloid infusion and 5 mg ephedrine. All hemodynamic measurements obtained after 30 min were stable, and we did not perform comparative analysis of these data. The incidence of nausea and vomiting did not differ significantly between the 2 groups (Table 2).

**Table 2:** Incidence of hypotension, nausea and vomiting among the two groups

	**Group A**	**Group P**	***P*-value**
	**(*n*** **= 40)**	**(*n*** **= 40)**	
Hypotension	12 (30%)	8 (20%)	0.30
Nausea and vomiting	3 (8%)	2 (5%)	1
Hallucination	0 (0%)	0 (0%)	1

Data are presented as number (%) of patients.

### Core body temperature

**Figure 4:**
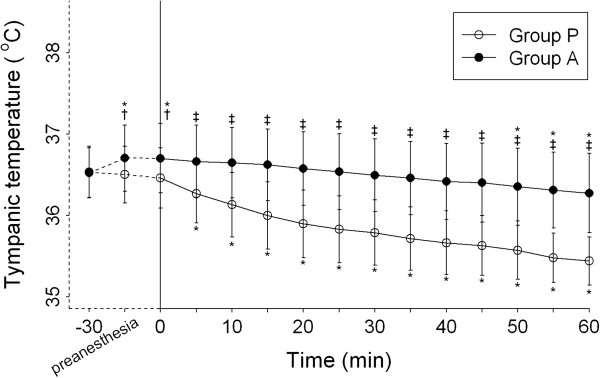
**Tympanic temperature before electroacupuncture, preanesthesia and after spinal anesthesia within 60 min. **Group P = placebo group, Group A = electroacupuncture group. Data are shown as mean (standard deviation). *P < 0.05 compared with baseline within groups; †*P* < 0.05 compared between groups; ‡*P* < 0.001 compared between groups.

The mean core temperature post-anesthesia compared with at baseline differed significantly between the 2 groups. The core temperature was significantly higher in Group A compared with Group P (*P* < 0.001) (Figure 4).

### Postanesthetic shivering

As Table 3 shows, 23 of 40 patients in Group P experienced shivering. This proportion was significantly higher than that found in Group A (13/40) (*P* = 0.03). Further analysis showed that in Group P, 10 of 40 patients experienced shivering at grade 4, whereas only 1 patient in Group A (1/40) experienced such severe shivering (Table 4). The difference between groups was statistically significant (*P* = 0.003).

**Table 3:** Number (%) of patients with shivering after 15 min of spinal analgesia

	**Group A (*n*** **= 40)**	**Group P (*n*** **= 40)**
Shivering *n* (%)	13 (33%)*	23 (58%)

Data are presented as number (%) of patients. Shivering refers to any shivering, i.e. Grades 1–4. *P = 0.03 compared with Group P.

**Table 4:** Number (%) of patients with different grades of shivering after 15 min of spinal analgesia

**Shivering score**	**Group A (*n*** **= 40)**	**Group P (*n*** **= 40)**
0	27 (68%)	17 (43%)
1	3 (8%)	1 (3%)
2	7 (19%)	9 (23%)
3	2 (5%)	3 (8%)
4	1 (3%)*	10 (25%)

Data are presented as number (%) of patients. *P = 0.003 compared with Group P.

For both groups combined, 20% of patients (16/80) experienced grade 3 or 4 shivering and requested treatment; these patients were administered 25 mg meperidine intravenously. After one such dose, shivering ceased in all patients.

## Discussion

Two main mechanisms of postanesthetic shivering are hypothermia and recalibration of the temperature setpoint to a higher level [1,18,19]. The management of postanesthetic shivering must focus on correcting these pathophysiologic changes [20-22], as preserving the patient’s body heat is an important issue. Butwick *et al*. [23] used a warming device to preserve body heat, and other physicians [9,20,21,24,25] have used drugs to constrict the blood vessels, thus preserving body heat.

The effect of drugs in preventing postanesthetic shivering has previously been investigated and compared [9,24,25]. To the best of our knowledge, ours was the first study to examine the application of electroacupuncture to prevent postanesthetic shivering during regional anesthesia. Our results (Table 3) showed that electroacupuncture exerted a significant antishivering effect compared with the placebo treatment. Furthermore, severe shivering (grade 4) was significantly lower in Group A than Group P (Table 4).

Lin *et al*. [26] proposed that stimulating acupoint ST36 may produce a slight increase in oral temperature, with a simultaneous decrease in the cutaneous temperature of the limbs (measurement limited to certain skin locations). The same researchers extended their study to record additional skin locations, and the results indicated that stimulation of ST36 on the left leg produced vasoconstriction in both legs, but not in either arm [13]. Reasons for the discrepant findings between the 2 studies were uncertain but may have included different needling depths (0.5 to 1.3 *cun* versus 0.5 to 2.3 *cun*). Dyrehag *et al*. [27] found that skin temperature tended to decrease after 30 min of electroacupuncture stimulation. This evidence supports the hypothesis that acupuncture to ST36 may lead to peripheral vasoconstriction.

In traditional Chinese medicine (TCM), ST36 is a *Xiahe* and *He* point of the stomach meridian of Foot-Yangming, and ST37 is a *Xiahe* point of the large intestine meridian. Clinical observation has shown that performing acupuncture on ST36 produces a clearer shape in the radial pulse. This finding may mean that the borders of the radial pulse emerge, which TCM physicians are able to detect by manipulation of the radial pulse through pushing or other movements. We speculated that this phenomenon may be attributed to peripheral vasoconstriction. We also hypothesized that acupuncture to ST36 may facilitate the preservation of heat. In addition, the Dao Ma needling technique, which is characterized by penetrating 2 to 3 adjacent acupuncture points simultaneously, is widely employed by Tung-style acupuncturists to enhance the therapeutic effect [28]. Based on these reasons, we chose the 2 acupuncture points of ST36 and ST37 for pretreatment of postanesthetic shivering.

Our results suggested that electroacupuncture to ST36 and ST37 could preserve core body temperature during regional anesthesia. According to our data, the mean tympanic temperature of patients increased slightly after 30 min of electroacupuncture treatment (before administration of spinal anesthesia), compared with baseline. In both the treatment and placebo groups, mean core temperature then gradually declined after anesthesia. However, this drop in temperature differed significantly between the 2 groups. The mean core temperature remained higher in Group A compared with Group P at the same time point (Figure 4).

Our findings indicated that acupuncture to ST36 and ST37 did not prevent bradycardia or hypotension after spinal anesthesia. The rate of bradycardia and hypotension (defined as a decrease in mean blood pressure of more than 20% from baseline) within 30 min after anesthesia did not differ significantly between groups. We also found no significant difference between the 2 groups for postanesthetic heart rate and mean blood pressure; in both groups, these 2 measurements declined step by step in a similar pattern. This result was similar to those of previous studies of drugs to prevent shivering [9,20,24,25]. The rate of nausea and vomiting, and of hallucination, did not differ significantly between the 2 groups (Table 2).

Previous studies have demonstrated that preoperative electroacupuncture on bilateral ST36 (Zusanli) acupoints with low and high frequency both can postpone time for the first dose of pethidine after operation and decrease the PCA demands and total morphine delivered in patients undergoing lower abdominal surgery [29]. Patients enrolled into the study underwent ureteroendoscopy, which is not major abdominal surgery, and suffered almost no pain in the operative room or postanesthetic room. There was no analgesic requirement for these patients. In view of this, it remains unknown whether electroacupuncture has led to pain-related phenomenon and side effects in our current study.

Previous studies have also shown that low frequency electroacupuncture at acupoints of lower extremities attenuates sympathetic nerve activity, which may mediate muscle shivering for heat production [30]. In this regard, low frequency electroacupuncture was selected as the treatment modality in the present study. In our pilot study setting, we adjusted the electric current based on the conception of patients. Current was tuned up to an amplitude that patient felt little but acceptable twitching, and then was tuned back to an amplitude that patient almost did not feel any twitching. We found that most patients did not feel any twitching with a current of 1 mA.

## Conclusions

Preservation of core body temperature is essential to prevent postanesthetic shivering, and electroacupuncture to ST36 and ST37 might achieve this objective. Although the precise mechanism is not yet clearly understood, electroacupuncture may work as a treatment modality for patients who experience shivering related to spinal anesthesia.

## Abbreviations

URS: Ureteroendoscopy; ASA: American Society of Anesthesiologists; Group P: Placebo group; Group A: Electroacupuncture group.

## Competing interests

The authors declare that they have no competing interests.

## Authors’ contributions

YCH and BYY designed the study protocol, drafted the manuscript and participated in the study as a coordinator. BYY participated in the study design and conducted the acupuncture treatment. CJC conducted statistical analysis. JYH, ITS, WJZ, YFT and HPY participated in the study design, drafted and reviewed the manuscript. All authors read and approved the final manuscript.

## Pre-publication history

The pre-publication history for this paper can be accessed here:

http://www.biomedcentral.com/1472-6882/12/233/prepub

## References

[B1] Ozaki M Kurz A Sessler DI Lenhardt R Schroeder M Moayeri A Noyes KM Rotheneder E Thermoregulatory thresholds during epidural and spinal anesthesia Anesthesiology 1994 81 282 288 10.1097/00000542-199408000-00004 8053576

[B2] Chan AM Ng KF Tong EW Jan GS Control of shivering under regional anesthesia in obstetric patients with tramadol Can J Anaesth 1999 46 253 258 10.1007/BF03012605 10210050

[B3] Sessler DI Ponte J Shivering during epidural anesthesia Anesthesiology 1990 72 816 821 10.1097/00000542-199005000-00008 2339797

[B4] Jeon YT Jeon YS Kim YC Bahk JH Do SH Lim YJ Intrathecal clonidine does not reduce post-spinal shivering Acta Anaesthesiol Scand 2005 49 1509 1513 10.1111/j.1399-6576.2005.00783.x 16223398

[B5] Alfonsi P Postanaesthetic shivering: epidemiology, pathophysiology, and approaches to prevention and management Drugs 2001 61 2193 2205 10.2165/00003495-200161150-00004 11772130

[B6] Kranke P Eberhart LH Roewer N Tramer MR Pharmacological treatment of postoperative shivering: a quantitative systematic review of randomized controlled trials Anesth Analg 2002 94 453 460 11812718 10.1097/00000539-200202000-00043

[B7] Kranke P Eberhart LH Roewer N Tramer MR Single-dose parenteral pharmacological interventions for the prevention of postoperative shivering: a quantitative systematic review of randomized controlled trials Anesth Analg 2004 99 718 727 10.1213/01.ANE.0000130589.00098.CD 15333401

[B8] Terasako K Yamamoto M Comparison between pentazocine, pethidine and placebo in the treatment of post-anesthetic shivering Acta Anaesthesiol Scand 2000 44 311 312 10.1034/j.1399-6576.2000.440316.x 10714846

[B9] Sagir O Gulhas N Toprak H Yucel A Begec Z Ersoy O Control of shivering during regional anaesthesia: prophylactic ketamine and granisetron Acta Anaesthesiol Scand 2007 51 44 49 10.1111/j.1399-6576.2006.01196.x 17229229

[B10] Fan CF Tanhui E Joshi S Trivedi S Hong Y Shevde K Acupressure treatment for prevention of postoperative nausea and vomiting Anesth Analg 1997 84 821 825 9085965 10.1097/00000539-199704000-00023

[B11] Crowley LJ Buggy DJ Shivering and neuraxial anesthesia Reg Anesth Pain Med 2008 33 241 252 18433676 10.1016/j.rapm.2007.11.006

[B12] Lin MT Chandra A Chen-Yen SM Chern YF Needle stimulation of acupuncture loci Chu-Chih (LI-11) and Ho-Ku (LI-4) induces hypothermia effects and analgesia in normal adults Am J Chin Med 1981 9 74 83 10.1142/S0192415X8100010X 7304501

[B13] Lin MT Chandra A Chen-Yen SM Effects of needle stimulation of acupuncture loci Nei-Kuan (EH-6), Tsu-San-Li (St-36), San-Yin-Chiao (Sp-6) and Chu-Chih (LI-11) on cutaneous temperature and pain threshold in normal adults Am J Chin Med 1981 9 305 314 10.1142/S0192415X81000408 7053029

[B14] Zhou W Longhurst JC Neuroendocrine mechanisms of acupuncture in the treatment of hypertension Evid Based Complement Alternat Med 2012 2012 878673 22216059 10.1155/2012/878673 PMC3246758

[B15] Andreescu C Glick RM Emeremni CA Houck PR Mulsant BH Acupuncture for the treatment of major depressive disorder: a randomized controlled trial J Clin Psychiatry 2011 72 1129 1135 10.4088/JCP.10m06105 21672495 PMC10536993

[B16] Tsai YC Chu KS A comparison of tramadol, amitriptyline, and meperidine for postepidural anesthetic shivering in parturients Anesth Analg 2001 93 1288 1292 10.1097/00000539-200111000-00052 11682416

[B17] Wilson E David A MacKenzie N Grant IS Sedation during spinal anaesthesia: comparison of propofol and midazolam Br J Anaesth 1990 64 48 52 10.1093/bja/64.1.48 2302376

[B18] Sessler DI Rubinstein EH Moayeri A Physiologic responses to mild perianesthetic hypothermia in humans Anesthesiology 1991 75 594 610 10.1097/00000542-199110000-00009 1928769

[B19] Buggy DJ Crossley AW Thermoregulation, mild perioperative hypothermia and postanaesthetic shivering Br J Anaesth 2000 84 615 628 10.1093/bja/84.5.615 10844839

[B20] Norouzi M Doroodian MR Salajegheh S Optimum dose of ketamine for prevention of postanesthetic shivering; a randomized double-blind placebo-controlled clinical trial Acta Anaesthesiol Belg 2011 62 33 36 21612143

[B21] Piper SN Rohm KD Maleck WH Fent MT Suttner SW Boldt J Dolasetron for preventing postanesthetic shivering Anesth Analg 2002 94 106 111 11772810 10.1097/00000539-200201000-00020

[B22] Miyawaki T Yao H Koyama E Maeda S Prevention of postanesthetic shivering with intravenous administration of aspirin J Anesth 1991 5 123 127 10.1007/s0054010050123 15278644

[B23] Butwick AJ Lipman SS Carvalho B Intraoperative forced air-warming during cesarean delivery under spinal anesthesia does not prevent maternal hypothermia Anesth Analg 2007 105 1413 1419 10.1213/01.ane.0000286167.96410.27 17959975

[B24] Mohta M Kumari N Tyagi A Sethi AK Agarwal D Singh M Tramadol for prevention of postanaesthetic shivering: a randomised double-blind comparison with pethidine Anaesthesia 2009 64 141 146 10.1111/j.1365-2044.2008.05711.x 19143690

[B25] Honarmand A Safavi MR Comparison of prophylactic use of midazolam, ketamine, and ketamine plus midazolam for prevention of shivering during regional anaesthesia: a randomized double-blind placebo controlled trial Br J Anaesth 2008 101 557 562 10.1093/bja/aen205 18621986

[B26] Lin MT Liu GG Soong JJ Chern YF Wu KM Effects of stimulation of acupuncture loci Ta-Chuei (Go-14), Nei-Kuan (EH-6) and Tsu-San-Li (St-36) on thermoregulatory function of normal adults Am J Chin Med 1979 7 324 332 10.1142/S0192415X79000295 543487

[B27] Dyrehag LE Widerstrom-Noga EG Carlsson SG Andersson SA Effects of repeated sensory stimulation sessions (electro-acupuncture) on skin temperature in chronic pain patients Scand J Rehabil Med 1997 29 243 250 9428058

[B28] Maher JH Advanced Tung Style Acupuncture 2005 RBC

[B29] Lin JG Lo MW Wen YR Hsieh CL Tsai SK Sun WZ The effect of high and low frequency electroacupuncture in pain after lower abdominal surgery Pain 2002 9 509 514 12406527 10.1016/S0304-3959(02)00261-0

[B30] Stener-Victorin E Jedel E Janson PO Sverrisdottir YB Low-frequency electroacupuncture and physical exercise decrease high muscle sympathetic nerve activity in polycystic ovary syndrome Am J Physiol Regul Integr Comp Physiol 2009 297 R387 R395 10.1152/ajpregu.00197.2009 19494176

